# Engineered mineralogical interfaces as radionuclide repositories

**DOI:** 10.1038/s41598-023-29171-1

**Published:** 2023-02-06

**Authors:** G. B. Douglas, S. M. Reddy, D. W. Saxey, C. M. MacRae, N. A. S. Webster, L. J. Beeching

**Affiliations:** 1 CSIRO Environment, Floreat, WA 6014 Australia; 2 School of Molecular and Life Sciences, Curtin University, Kent St, Bentley, WA 6102 Australia; 3 School of Earth and Planetary Sciences, The Institute for Geoscience Research (TIGeR), Curtin University, Bentley, WA 6102 Australia; 4 Geoscience Atom Probe, John de Laeter Centre, Curtin University, Bentley, WA 6102 Australia; 5 CSIRO Mineral Resources, Clayton, VIC 3168 Australia

**Keywords:** Geochemistry, Nanoparticles

## Abstract

Effective capture of fugitive actinides and daughter radionuclides constitutes a major remediation challenge at legacy or nuclear accident sites globally. The ability of double-layered, anionic clay minerals known as hydrotalcites (HTC) to contemporaneously sequester a range of contaminants from solution offers a unique remedy. However, HTC do not provide a robust repository for actinide isolation over the long term. In this study, we formed HTC by in-situ precipitation in a barren lixiviant from a uranium mine and thermally transformed the resulting radionuclide-laden, nanoscale HTC. Atomic-scale forensic examination of the amorphized/recrystallised product reveals segregation of U to nanometre-wide mineral interfaces and the local formation of interface-hosted mineral grains. This U-phase is enriched in rare earth elements, a geochemical analogue of actinides such as Np and Pu, and represents a previously unreported radionuclide interfacial segregation. U-rich phases associated with the mineral interfaces record a U concentration factor of ~ 50,000 relative to the original solute demonstrating high extraction and concentration efficiencies. In addition, the co-existing host mineral suite of periclase, spinel-, and olivine-group minerals that equate to a lower mantle, high P–T mineral assemblage have geochemical and geotechnical properties suitable for disposal in a nuclear waste repository. Our results record the efficient sequestering of radionuclides from contaminated water and this novel, broad-spectrum, nanoscale HTC capture and concentration process constitutes a rapid solute decontamination pathway and solids containment option in perpetuity.

## Introduction

Uranium, associated ore elements, and daughter radionuclides, liberated in mining, beneficiation, nuclear fission and waste management, encompass a diversity of chemical reactivity and half-lives that are environmentally hazardous and constitute a pervasive challenge for safe disposal into the millennia^1,2^. While technologies have been investigated to capture U^3–5^, or radioactive ^137^Cs, ^129^I, or ^90^Sr, often in isolation^6–8^, few technologies can reliably sequester and retain a suite of radionuclides.

The formation of layered-double hydroxide (LDH) minerals, specifically hydrotalcite (HTC), an Mg–Al rich LDH form ([Mg_1−x_Al_x_](OH)_2_A_x/m_−^m−^·nH_2_O, A = anion), has previously been investigated as a solid phase, *pre-formed* sorbent for U removal over a range of pH, ionic strength, and in the presence of co-existing U ligands^9–15^. Nanoscale polymetallic HTC formed in situ in wastewaters via a bespoke, self-assembly mechanism may incorporate U, daughter radionuclides and other contaminants as fundamental building blocks within or at the edges of the metal hydroxide and interlayers, respectively^16–22^. However, the resulting HTC comprises nanometric crystallites and agglomerates that may be unstable during exposure to natural environmental conditions over yearly timescales^23^. Thus, while broad spectrum contaminant uptake via in situ precipitation has been demonstrated, a challenge exists to further isolate and concentrate U and other radionuclides, whether for separation or alternatively, as a means to generate a robust mineral-based repository for long-term disposal.

Knowledge of the mineralogical distribution and partitioning of radionuclides within a repository material is essential for a thorough assessment of long-term stability. This nanoscale information is typically challenging to acquire, with conventional techniques ranging from selective chemical extraction to SEM and synchrotron-based X-ray fluorescence methods frequently utilised. These techniques, however, have inherent spatial-resolution limitations that often preclude the differentiation of radionuclide location relative to the bulk matrix. This limits knowledge of the potential geochemical reactivity in the context of suitability for disposal in a long-term nuclear repository. Consequently, geochemical modelling is often utilised in combination with instrumental and chemical techniques or as a surrogate^24^.

In this contribution, we specifically address these diverse characterisation challenges by nanoscale analysis of the products of a combined HTC in situ precipitation and thermal decomposition process^22^. This HTC-based capture and recovery approach typically involves selective addition of Mg- and/or Al-containing compounds to obtain a suitable M^2+^:M^3+^ cation stoichiometry to form HTC within a radionuclide-bearing wastewater^16–22^. Thereafter, potential addition of other compounds such as silica or aluminosilicates, followed by thermally-mediated decomposition over a calcination-cooling cycle enables the formation of a suite of secondary minerals that facilitate selective fractionation and concentration of U, radionuclides and rare earth elements (REE), the latter of which can be considered as actinide analogues.

Here we synthesize HTC in situ from an acidic, oxidising barren lixiviant from the Beverley North Uranium mine, South Australia, containing U and a diverse suite of daughter radionuclides, REE and other elements^16^, and thermally recrystallise the resulting nanoscale HTC product by heating to 1320 °C. We investigate the mineralogical evolution of the recrystallising material by thermal XRD and the resulting nanoscale distribution and partitioning of U, daughter radionuclides and environmentally-significant contaminants are assessed by correlative integration of scanning electron microscopy (SEM), electron backscatter diffraction (EBSD), transmission electron microscopy (TEM), high-angle annular dark-field-scanning transmission electron microscopy (HDAAF-STEM) and atom probe tomography (APT).

## Results

### Hydrotalcite formation

**Figure 1:**
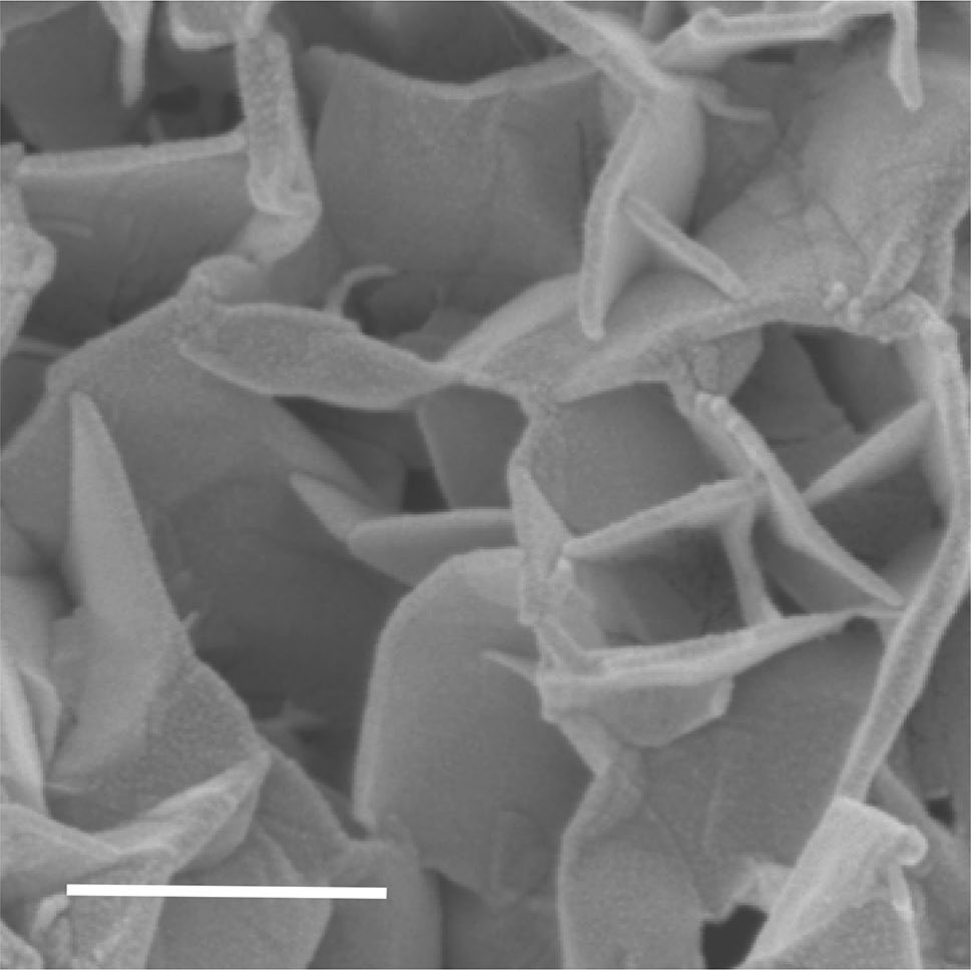
Aggregated hydrotalcite (HTC) platelets formed in situ from a U mine barren lixiviant. Scale bar is 500 nm.

In situ HTC formation from a the acidic, oxidising U mine barren lixiviant^16^ resulted in the formation of botryoidal aggregates typically 10–30 µm in diameter. Within these aggregates hexagonal HTC platelets of ~ 0.5 to 1.0 µm in diameter and 0.05 to 0.1 µm in thickness were irregularly packed in a face to edge or edge to edge arrangement (Fig. 1). Mineralogical (XRD) data confirmed the presence of a monomineralic precipitate, with XRF analysis indicating that the HTC had a ~ 3:1 M^2+^:M^3+^ stoichiometry^16^. Substantial U-series radionuclides were contained in the HTC with a total activity of ~ 2.8 M Bq kg^−1^.An Extended X-ray Absorption Fine Structure (EXAFS) study identified that U is predominantly associated with the surface of the HTC as inner-sphere complexes^21^.

### XRD, SEM and TEM analysis

**Figure 2:**
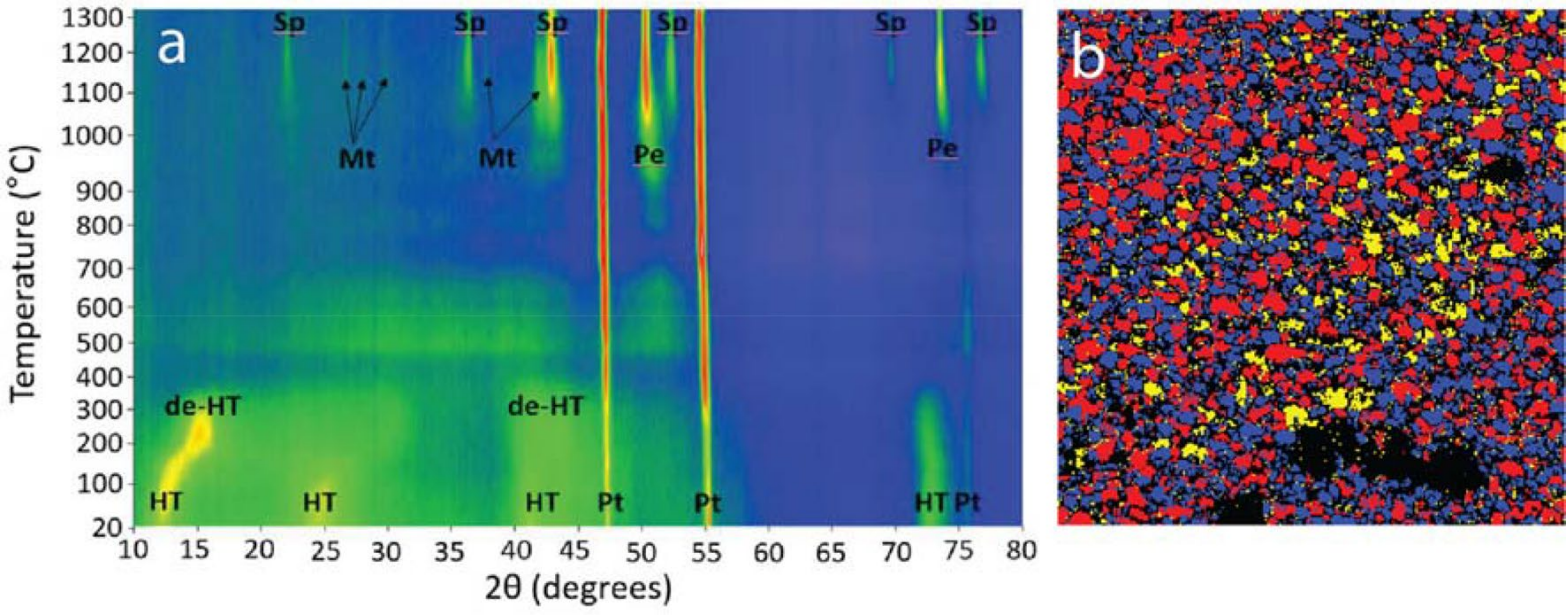
(**a**) Evolution of X-Ray diffraction signal during amorphization and subsequent phase transformation of hydrotalcite as a function of temperature (20–1320 °C). *HT* hydrotalcite, *de-HT* dehydrated hydrotalcite, *Pe* périclase, *Sp* spinel, *Mt* monticellite. Pt represents the platinum heating strip within the experimental cell. (**b**) EBSD phase map confirming the mineralogy as determined in (**a**) and showing the distribution and textural relationships among spinel (red), periclase (blue) and monticellite (yellow).

**Figure 3:**
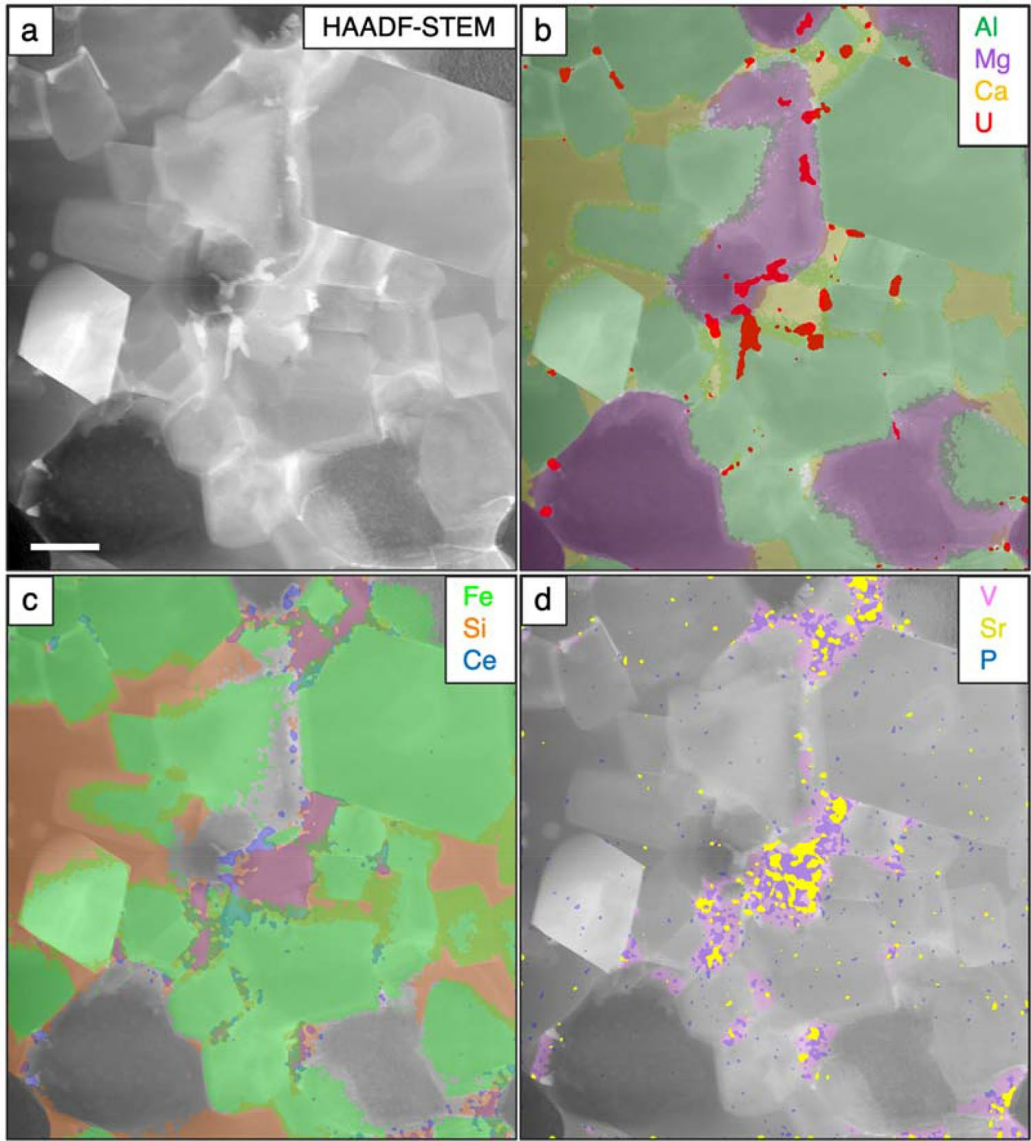
HAADF-TEM images (**a**) greyscale, (**b**) false color element images of Al (green), Mg (purple), Ca (yellow) and U (red), (**c**) false color element images of Fe (green), Si (orange) Ce (blue), and (**d**) V (pink), Sr (yellow) and P (blue)—where P and Sr are coincident, P appears as a pale purple. Scale bar in (**a**) is 500 nm.

XRD data collected during the heating experiment record the amorphization and subsequent phase transformation of HTC to the higher temperature minerals, predominantly spinel and periclase, and lesser of the olivine-group mineral, monticellite (Fig. 2a). Both SEM (Fig. 2b) and TEM (Fig. 3a–d) imaging reveal three major mineral phases within the thermally modified HTC. EBSD data confirms the identity of the three major phases determined from the XRD analysis. SEM- and TEM-derived EDS maps (Fig. 3b–d respectively) constrain the compositions of the euhedral spinel (Fe–Al–Mg), the subhedral periclase (Mg), and the anhedral monticellite (Ca–Si), which is found as a localised, interstitial phase (Figs. 2b, 3a–d).

### APT analysis

**Figure 4:**
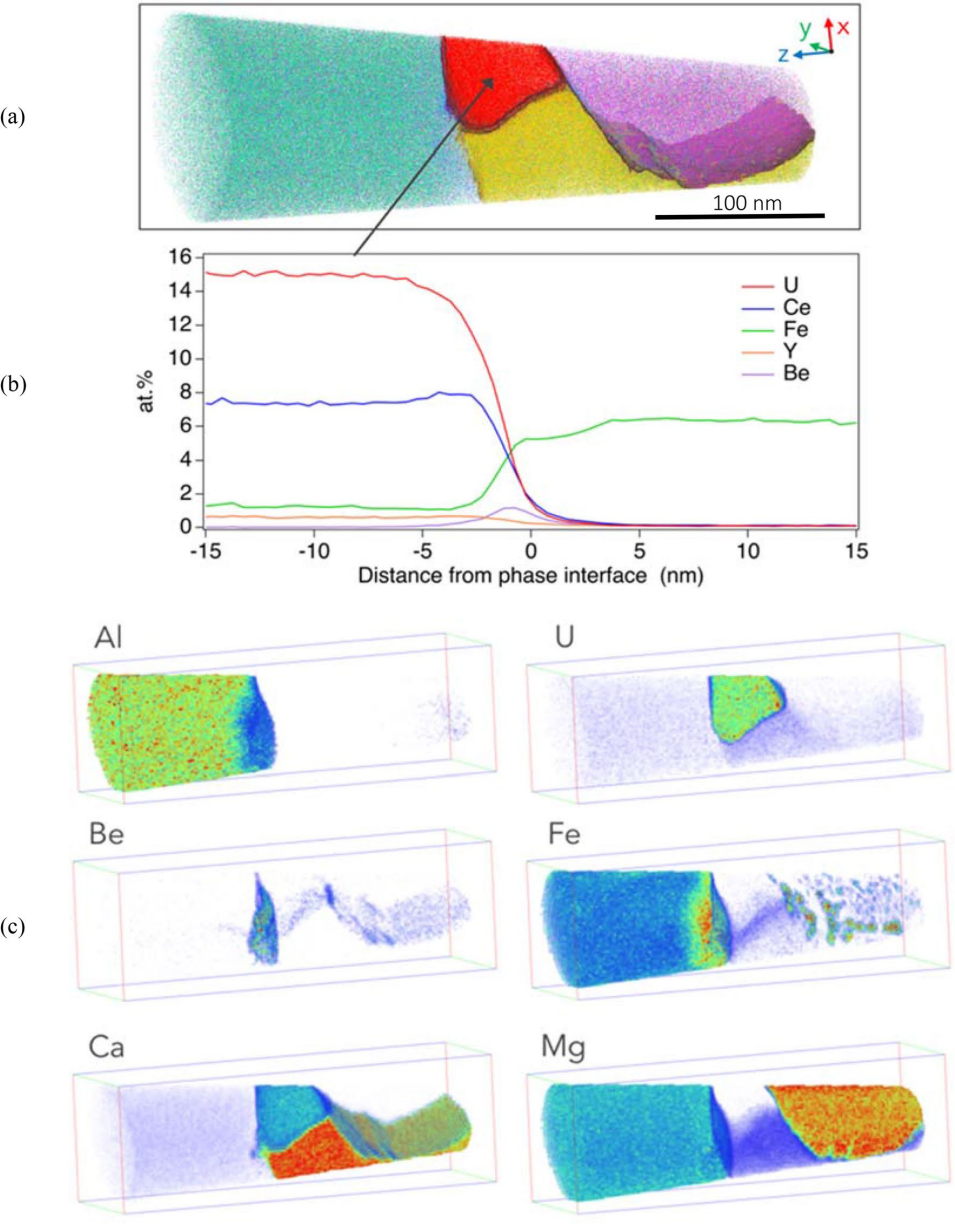
APT analysis of specimen M22 (**a**) 3D reconstructed atom map of a multiphase region within APT specimen. Atoms are displayed for Mg (purple), U and Ce (red), Ca and V (yellow), Fe and Al (green), Be (blue). Iso-concentration surfaces correspond to 11 at.% U (red), 20 at.% Mg (purple), and 30 at.% Ca (yellow). The reconstructed volume is ~ 300 nm in length with the images compiled from ~ 34 M atoms. (**b**) A proximity histogram, or proxigram, showing the element profile from the U-REE phase, in all directions across the phase boundary as defined by the 11 at.% U iso-concentration surface. (**c**) 3D renderings of selected element concentrations using a color/transparency scale.

**Figure 5:**
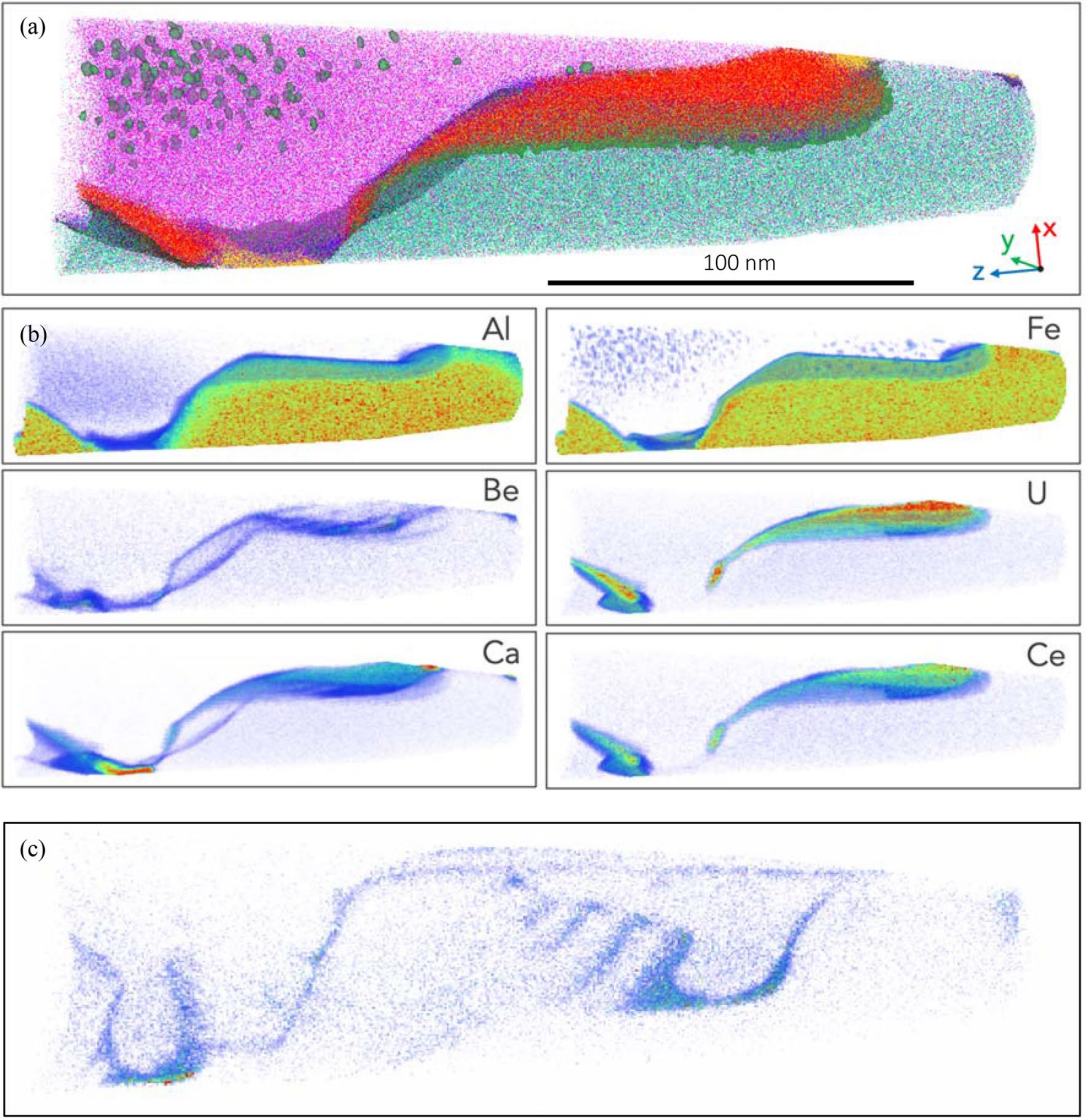
(**a**) APT analysis of specimen 1592 (**a**) 3D reconstructed atom map of a multiphase region within APT specimen. Atoms are displayed for Mg (purple), U and Ce (red), Ca and V (yellow), Fe and Al (green), Be (blue). Iso-concentration surfaces correspond to 11 at% U (red), 20 at% Mg (purple), and 30 at% Ca (yellow). The reconstructed volume is ~ 300 nm in length with the images compiled from ~ 34 M atoms. (**b**) 3D renderings of selected element concentrations using a color/transparency scale. (**c**) Volume rendered image of the Be complexion rotated 50 degrees to highlight the complex interfacial association along adjacent mineral surfaces.

The composition of the three major phases are further constrained by APT analysis (Table 1). In addition, APT shows spherical Fe-rich clusters (< 10 nm diameter) within the periclase. These are largely absent from the periclase grain margins (Figs. 4, 5) and are interpreted to represent exsolution of an Fe-rich oxide.

**Table 1:** Radionuclide, major and trace element partitioning between U-REE oxide, pliniusite, monticellite, spinel and periclase (expressed in at.%) determined by Atom Probe analysis.

Elements	U-REE oxide	Pliniusite	Monticellite	Spinel	Periclase
Radionuclides (wt%)					
U	14.66	0.01	–	–	–
Th	1.36	0.05	–	–	–
^ 210^Pb	0.02	–	–	–	–
REE (wt%)					
Total REE	9.67	0.28	–	–	–
Major element oxides (wt%)					
Si	–	2.40	14.82	-	0.03
Al	–	–	0.09	22.82	0.46
Fe	1.52	–	–	9.90	3.23
Mg	0.50	1.92	20.10	17.20	45.53
Ca	18.30	43.59	16.63	0.01	0.01
P	-	0.91	0.01	–	–
Trace elements (wt%)					
Sr	0.32	1.18	–	–	–
V	0.38	11.99	0.02	–	–

High spatial resolution imaging and compositional analysis further identifies two additional phases (Fig. 3). A subhedral Ca + V rich phase is spatially associated with monticellite, but is found in the interstices between the major phases. Individual grains record irregular, intragranular variations in V, Sr and P (Fig. 3d). The composition of this phase, as determined by APT (Table 1), is consistent with an apatite group mineral associated with the pliniusite: Ca_5_(VO_4_)_3_F)—apatite (Ca_5_(PO_4_)(OH,F,Cl) solid solution series^25^. Further associated with this phase are irregularly shaped grains of a U + REE oxide (Fig. 3, Table 1).

**Figure 6:**
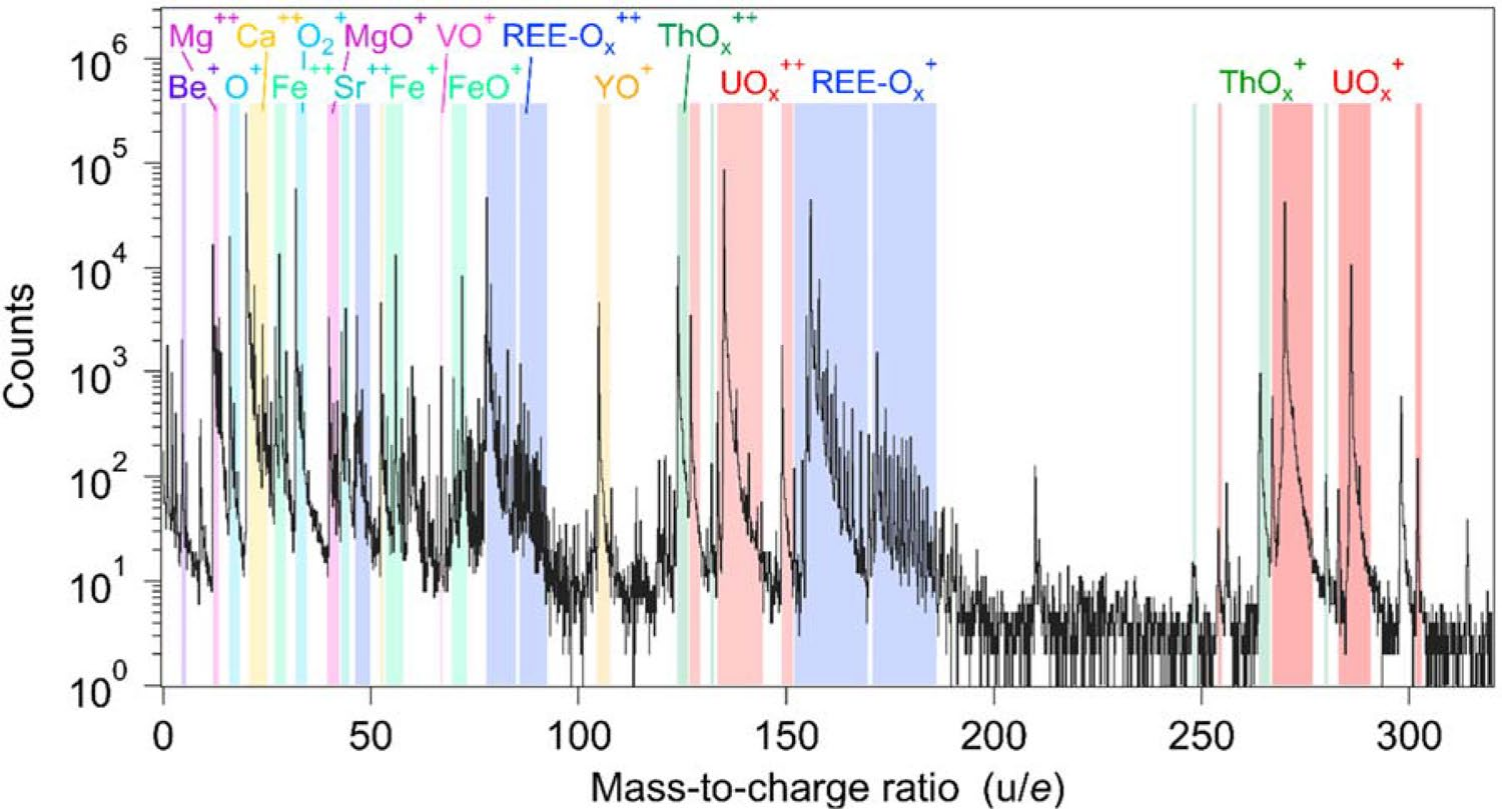
Atom probe tomography (APT) selected mass spectrum for the U-REE phase. Major ionic peaks are identified.

APT characterisation of the U-REE oxide show two discrete textural positions; either as small ~ 50 nm, interface-hosted grains (Fig. 4) or as a segregated phase (< 10 nm wide) along grain boundaries (Fig. 5). In addition to U and REE, these interfaces are also enriched in Th and Be (Figs. 3a,b, 5, 6), with Be defining equally-spaced, linear features interpreted as dislocations (Fig. 5c).

## Discussion

Capture and segregation of radionuclides and other contaminants to nanoscale phases and mineral interfaces is accomplished by two distinct reaction sequences. Firstly, a composition- and pH-mediated self-assembly of polymetallic HTC nanocrystals facilitated by the stoichiometric addition of M^2+^ and/or M^3+^ cations^16^. With increasing pH, a cascade of radionuclide and other contaminant metal ions are progressively incorporated into the evolving HTC structure, as their respective metal hydroxide solubilities are exceeded^16–22^. Anions are correspondingly incorporated within the positively-charged, metal hydroxide HTC interlayers. Once formed, spontaneous aggregation and settling of HTC grains readily separates them from their co-existing solute.

**Figure 7:**
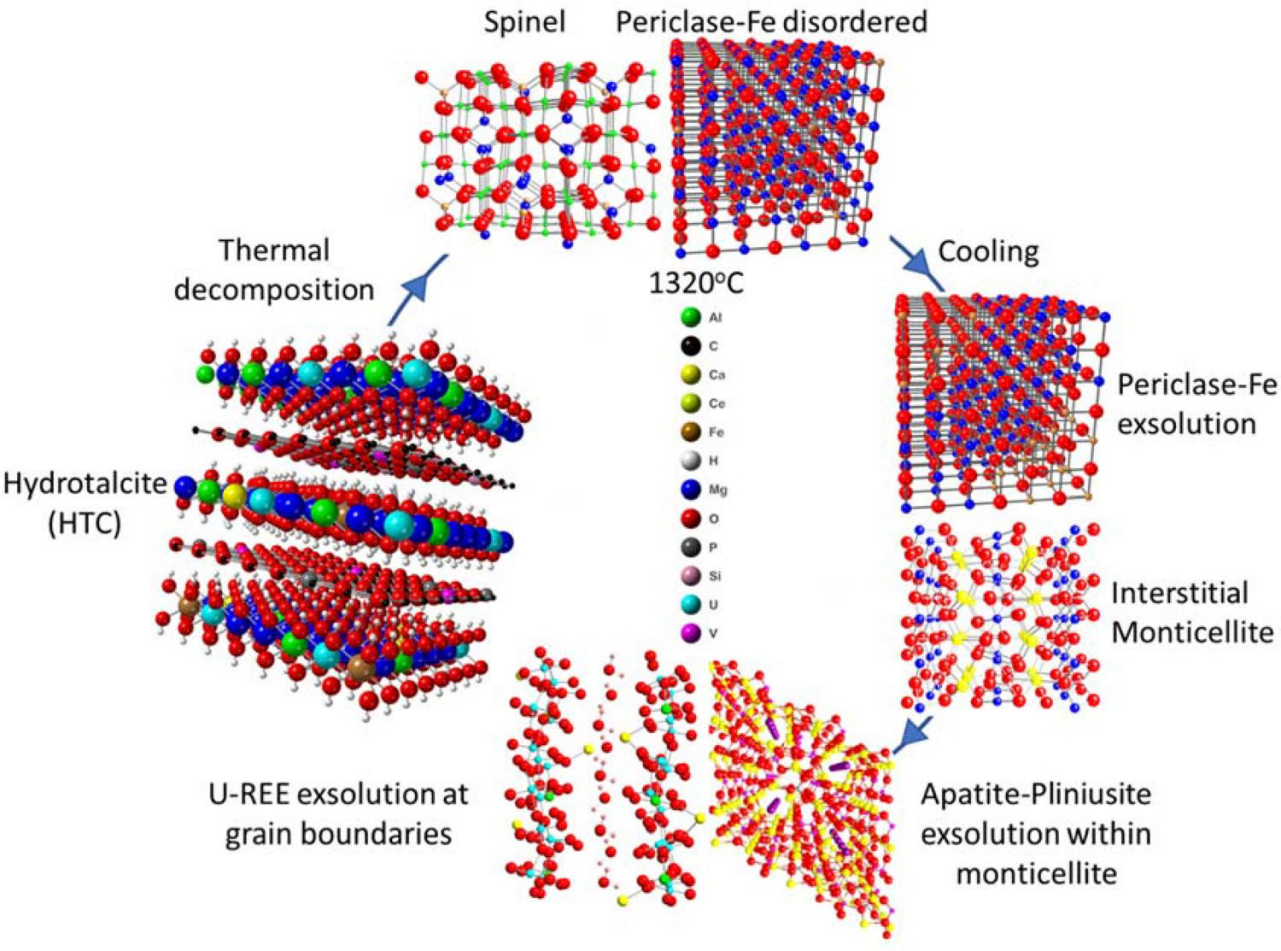
Visualization of polymetallic hydrotalcite (HTC) structure with mixed metal hydroxide layer and interlayer occupancy, its amorphisation/recrystallisation into ferropericlase and spinel followed by cooling with the interstitial crystallisation of monticellite, Fe exsolution in the periclase host, and the exsolution of both apatite-pliniusite within monticellite and a U-REE phase along periclase and spinel intergranular boundaries.

The second stage involves calcination and thermally-mediated amorphization and subsequent phase transformation of the HTC up to 1320 °C (Figs. 2, 7). In the initial stages of heating from 150 to 350 °C, HTC undergoes dehydration and dehydroxylation resulting in the formation of a predominantly Mg–Al double oxide phase (Fig. 2). Above 350 °C, XRD data (Fig. 2) indicate a poorly crystalline material as mixed metal, predominantly Mg–Al oxides, which appear largely amorphous until 850 °C, where it commences formation of discrete mineral phases. These involve the formation of periclase and spinel, and later, interstitial monticellite as the three major sub-volumes. Both the periclase and monticellite exhibit increasing crystallinity with higher temperature. A similar reaction sequence has also been reported in model HTC systems^26^.

Upon cooling, the Fe present in the periclase undergoes intra-phase exsolution (Figs. 4, 7), likely due to prohibitive mineral lattice parameter constraints, forming discrete intra-host Fe oxides. The absence of these Fe clusters within ~ 50 nm of the grain margin indicates the ability of Fe to diffuse to the grain boundary over these length scales.

Large ion lithophile elements (LILE) such as U and the REE were potentially accommodated at elevated temperatures within expanded lattice sites. With decreasing temperatures, the absence of a suitable host mineralogy resulted in the segregation of these elements to the mineral interfaces to produce interfacially-hosted mineral grains and films^27^ (Fig. 3), most notably U and REE; and V and REE into pliniusite apatite with vanadate for phosphate oxyanion substitution (Fig. 7).

We have demonstrated that an integrated HTC formation/thermal amorphisation and subsequent phase transformation process provides a multi-radionuclide and contaminant capture and containment protocol that has previously been deployed at scale for acidic^16,20^ to circumneutral^28^ metal and metalloid contaminated waters. On this basis, the technology described in this study had the potential be utilised for the capture and remediation of radionuclide contaminated wastewaters from nuclear incidents such as in the immediate aftermath of the Fukushima Daiichi Nuclear Power Plant (FDNPP) incident, and during on-going remediation efforts in both surface (reactor and tank) and subsurface (groundwater) domains.

In addition to the capture of solubilised ^235^U fuel, a suite of important nuclear fission radionuclides, e.g. ^90^Sr (t_1/2_: 28.8 d) and ^140^Ba (t_1/2_: 12.8 d)^29^ and anionic/complex radionuclides including ^129^I (t_1/2_: 15.7 M year) and ^99^Tc (t_1/2_: 211 K year), may also be captured within HTC metal hydroxide layers^29,30^. Total REE captured from the barren lixiviant is concentrated to ~ 9.7 at.% within the interfacial U-bearing phase which also contains ~ 14.7 at.% U and ~ 1.4 At % Th and 0.02 at.% ^210^Pb (Table 1) confirming the propensity of this phase to simultaneously host a range of radionuclide contaminants. The REE, with + II (Eu^2+/3+^) to + IV (Ce^3+/4+^) oxidation states, where in the U-REE phase the Ce constitutes ~ 80 at.% of the total REE, are considered as proxies for actinides including Np and Am, indicating that substantial actinides could be similarly accommodated, initially within the HTC, and then following calcination, further concentrated within interfacial mineral phases.

Radionuclide concentration factors are an important aspect to consider given the potential of the use of the method described here as a pathway for long-term radionuclide disposal. The in-situ formation of HTC in this study yielded ~ 3 g/L (0.3 wt%) of precipitate, with ~ 330 times concentration factor of radionuclides and other contaminants over the solute. Thermal decomposition of the HTC yielded a further ~ 35% solid mass reduction due to dehydration/dehydroxylation, with a consequent ~ 1.5 times concentration factor (CF). Thereafter, HTC phase transformation and interfacial segregation resulted in sub-volumes estimated to be < 1%, and an additional ~ 100 times CF. Thus, the aggregate concentration factor for U and associated radionuclides within interfacial segregations is ~ 50,000 CF over initial solute concentrations. With an initial activity of 12,630 Bq/L in the Beverley North mine barren lixiviant^16^, and assuming radionuclide quantitative uptake, segregated U and daughter radionuclides could attain an aggregate activity of ~ 10^8^ Bq/kg contained within ~ 0.002% of the initial solute volume. Applying similar concentration factors to the specific context of Fukushima^31,32^, and using current measurements of Fukushima tank farm activities^33,34^, we estimate average HTC activity would be in the order of ~ 1.4. × 10^6^ Bq/g, increasing to ~ 2.1 × 10^8^ Bq/g following phase transformation and interfacial segregation.

Following removal of radionuclides from contaminated waters, a challenge remains regarding long-term storage. Nuclear repository materials require chemical, thermal, and geotechnical properties that mitigate against radionuclide remobilisation and exhibit resistance to structural damage/amorphization from radioactive, and in particular, alpha decay^35^. The high radionuclide/contaminant loading capacity of HTC, and ease of separation, coupled with a single thermal amorphization/phase transformation step, will maximise recovery and minimise processing loss. In contrast to additive, cementation technologies for the immobilisation of radioactive waste, the processing pathway via HTC and amorphization and phase transformation does not substantially increase final wasteform mass. Thus, HTC precipitation and thermally-mediated amorphization and phase transformation may also constitute a viable, alternative synthetic pathway for other radionuclide-contaminated waters (e.g. raffinate^36^ or mixed waste/sludge vitrification/immobilisation) and conventional waste management strategies involving silicate-based glass (e.g. Sellafield^37^ and Hanford^36–39^).

In this study, the successful capture, concentration and retention of radionuclides and formation of interfacial U-REE phases is associated with the crystallisation of spinel, periclase and olivine group minerals typical of a lower mantle (high P–T) assemblage. Both periclase and spinel are inherently tolerant to radiation-induced amorphization^40–42^ and hence, the potential release of contained radionuclides. These two phases constitute ~ 95% of the recrystallised HTC mineralogy. Both periclase and spinel confer high temperature strength and thermal stability and low volumetric expansion at 1000 °C of ~ 2% and 1.5%, respectively^43^, consistent with their occupancy in the lower mantle and essential prerequisites for a long-term nuclear repository material. Notably, periclase is currently used as an engineered barrier material in waste repositories and is the only material certified by the US EPA for emplacement in the Waste Isolation Pilot Plant (WIPP)^44^. A similar brucite-based engineered barrier has been employed in the German Asse repository^45^.

In the context of a long-term nuclear repository setting, periclase is fortuitously both a strong acidity buffer^46^ in the event of an influx of acidic groundwater or interaction with other acid-forming nuclear repository components, and a CO_2_ scavenger via magnesite (MgCO_3_) formation under high T, and potentially P–T conditions within a nuclear repository. Importantly, periclase reactivity is mediated by the formation of a passivating surface brucite layer, thus constituting a self-healing, reflexive pH buffer system. Coincidentally, with the reaction of the periclase, and in the presence of other M^2+^ or M^3+^ cations, formation of nanoscale secondary hydroxide phases including HTC may also occur, analogous to the in-situ reaction initially used to decontaminate the radionuclide-bearing solutes.

Importantly, stoichiometric addition of silica to the in-situ formed HTC prior to calcination could be used to facilitate the formation from periclase of predominantly olivine, mixed olivine-pyroxene or pyroxene suites of minerals where periclase could be concerted to olivine (forsterite) and pyroxene (enstatite) with progressive (stoichiometric) silica addition, with existing monticellite converted to a Ca-Mg pyroxene (diopside). This compositional and mineralogical flexibility offers a range of alternative final repository mineralogies that may be tailored to be tolerant to radiation damage^40–42^ from specific radionuclides or combinations thereof.

The formation of pliniusite is comparable to apatite synthesised in conventional nuclear repository materials, and thus can be considered as a ^90^Sr repository. The pliniusite may also act as a repository for ^129^I as mineralogical analogue of fluorapatite^47^.

In the wider context of the efficient, broad-spectrum capture and retention of radionuclides over millennia, the application of in-situ HTC formation confers a number of important physico-chemical and operational advantages for the decontamination of current and legacy nuclear contaminated waters. This study assumes global importance when considered in the context of nuclear accidents at Chernobyl (1986) and Fukushima (2011) which highlight the need for a rapid, simple to deploy, multi-radionuclide capture and containment technology to mitigate against release of radionuclides in reactor wastewaters, or that already released to local surface or groundwater. The presence of U-rich complexions along mineralogical grain boundaries as formed in this study also provides a fundamentally new mineralogical architecture for post-fission radionuclide capture and stabilization as part of long-term nuclear waste repository confinement strategies. The additional combination of thermally-mediated amorphization/phase transformation, and a tuneable, intrinsically periclase and spinel, lower mantle-like mineralogy hosting a radionuclide-enriched, interfacial minerals, constitutes a promising new disposal pathway for radionuclide-contaminated solids management in perpetuity.

## Methods

### Hydrotalcite (HTC) sample preparation

The HTC was synthesized from an acidic, oxidising barren lixiviant from the Beverley North Uranium mine using conditions described elsewhere^16–18^. Briefly, this is as follows: a two-litre sample of barren lixiviant (activity 12,630 Bq/L) was placed in acid-washed Erlenmeyer flask and stirred rapidly using a Teflon-coated magnetic stirrer bar. Laboratory grade MgCl_2_–6H_2_O (13 g) required to achieve an Mg:Al molar stoichiometry of 3:1 was added in a single action as a dry powder. Immediately thereafter, NaOH (1 M) was added drop wise to a pH 10 endpoint. The solution was then further stirred for ~ 0.5 h. The HT suspension was then aged with the co-existing supernatant at 50 °C in a laboratory oven for seven days, the supernatant decanted, washed repeatedly with deionised water and oven-dried at 50 °C.

### XRD sample preparation/thermal decomposition/amorphization and phase transformation

The HTC sample was heated over the range 20–1320 °C at a rate of 20 °C min^−1^ under a flow of air. Variable-temperature X-Ray Diffraction (XRD) experiments were performed on the HTC sample using an INEL diffractometer, which incorporates a CPS120 position sensitive detector allowing for simultaneous collection of up to 120° 2θ of diffraction data using a Co tube operated at 40 kV and 35 mA. An Anton Paar HTK10 high-temperature chamber employing a Pt resistance strip heater containing a sample well measuring ~ 20 × 7 × 0.2 mm was fitted to the diffractometer. The XRD data were collected over the range 10° ≤ 2θ ≤ 120° continuously throughout heating, with individual datasets collected for one minute under the ambient atmosphere.

### Post-thermal amorphisation/phase transformation sample analysis

Reaction products from the XRD analysis/thermal amorphisation/phase transformation were mounted in an epoxy resin button for subsequent Scanning Electron Microscopy (SEM), Electron Dispersive Spectroscopy (EDS) chemical mapping, Electron Backscatter Diffraction (EBSD) mapping, and specimen preparation for Atom Probe Tomography (APT) and Transmission Electron Microscopy (TEM). All electron microscopy and atom probe analyses were carried out within the John de Laeter Centre, at Curtin University, Perth, Western Australia.

### SEM imaging and mapping

A Tescan Lyra3 Focussed Ion Beam SEM (FIB-SEM) was used to image and map the mounted samples^47^. With an electron beam energy of 20 kV, wide-field images were generated using the backscattered electron (BSE) signal to view the large grains of the major mineral phases. Higher-resolution BSE scans were used to locate and image phases enriched in heavy elements. EDS mapping was performed over ~ 100 × 100 µm^2^ areas, using a 30 kV electron beam, to further characterise the chemistry of the visible grains and to allow targeting of specific regions for APT and TEM specimen preparation.

### Electron backscatter diffraction

EBSD mapping was performed using a Tescan MIRA3 Field Emission SEM, with an Oxford Instruments AZtec acquisition system that has combined energy dispersive X-ray (EDX) and EBSD capability. The data were collected at 20 kV, a beam current of ~ 1 nA, and a working distance of 20 mm. The sample was tilted at 70° and EBSD data were collected in map form covering ~ 2500 µm^2^ and the step size used for data collection was 200 nm. Collected electron backscatter diffraction patterns were indexed by reference to crystallographic data for periclase, spinel, and monticellite. Post-processing of EBSD data to produce phase and orientation maps was undertaken in Oxford Instruments Channel 5.12 software. Following standard EBSD protocols, a wildspike and a 6 nearest neighbour noise reduction was undertaken on the data.

### Atom probe tomography

Samples from the reaction products were prepared for analysis by Atom Probe Tomography (APT), which provides three-dimensional nanoscale chemical mapping. APT involves laser-assisted, field-evaporation of individual and complex ions from the apex of a needle-shaped specimen. Time-of-flight mass spectroscopy is used to identify the mass/charge ratio of ions or molecular species and spatial information is obtained from the ion flight paths and the impact location on a position-sensitive detector^48–53^.

Targeted specimen preparation relies upon the positioning of a feature of interest within the central 100 nm of a needle specimen, and within a few hundred nm of the apex. For this study, we followed a well-established approach for the extraction, mounting and preparation of atom probe specimens^48^ which has been slightly modified to allow easier targeting of small regions of interest. Targeted regions were selected from bright areas on the BSE image.

A Tescan Lyra3 FIB-SEM was used to undertake site-specific targeting of the heavy element-rich phases identified by correlative techniques (SEM, EBSD). Wedge-shaped lift-outs were cut using a 30 kV Ga^+^ ion beam, and were extracted from the sample using an *in-situ* nanomanipulator (SmarAct). The wedge was sliced and mounted on a series of pre-fabricated Si posts and sharpened into a needle shape using annular milling, again using a 30 kV Ga^+^ focused ion beam. Finally, a 2 kV beam was used in order to remove most of the Ga^+^ ion damage from the specimen apex. The resulting specimen needles had a tip radius of ~ 100 nm.

APT data acquisition was performed in pulsed-laser mode on a Cameca LEAP 4000X HR atom probe microscope using a laser with wavelength of 355 nm. The laser pulse energy was set between 100 and 150 pJ with a pulse rate of 125 kHz, to allow a sufficient time-of-flight window for the high-mass ions evaporated during the analyses. For the analyses, the target ion detection rate was set to 1.0–1.2%, with the base temperature set at 60 K.

The APT data sets were reconstructed and analysed using the Cameca Integrated Visualization and Analysis Software package (IVAS), version 3.8. Peaks within the mass spectrum were identified and quantified by assigning spectral ranges across the full peak width visible above the background. Spatial reconstructions were performed using a constant shank angle assumption to provide a more consistent spatial representation of the data in the presence of multiple mineral phases. Isoconcentration surfaces were used to isolate the various phases present in the APT datasets, and chemical profiles across phase boundaries were generated using the proximity histogram, or proxigram method^51^. Compositional information was calculated by decomposition of molecular ion counts and using background-corrected values generated within IVAS.

### Transmission electron microscopy

Transmission Electron Microscopy (TEM) specimens were prepared with a Tescan Lyra3 FIB-SEM, using an *in-situ* lift-out technique to target the regions of interest. A 30 kV Ga^+^ ion beam was used to excavate and thin the lamella specimen. A 2 kV beam was used in the final step to remove most of the surface layers affected by Ga^+^ ion damage. TEM imaging and analysis was performed with an FEI Talos FS200X FEG TEM, using a 200 kV beam energy. EDS maps were collected in scanning-TEM (STEM) mode, using a ‘Super-X’ EDS detector with a large angular field-of-view. High-angle annular dark field (HAADF) images were collected in STEM mode with the ‘DF4’ annular detector. The raw compositional (EDS) maps were processed using the GIMP 2.10 software package, with filtering and thresholding applied to produce composite maps (Fig. 3a–d).

## Supplementary Information

Supplementary Information.

## Data Availability

Supplementary Information for Fig. 4b contained in Supplementary Information: SI Fig.4b_Proxigram_data.xlsx. The datasets used and/or analysed during the current study available from the corresponding author on reasonable request.
