# Supplementary material for: Engineered mineralogical interfaces as radionuclide repositories
Source: Sci Rep. 2023 Feb 6;13:2121. doi: 10.1038/s41598-023-29171-1 (PMC9902532; doi:10.1038/s41598-023-29171-1)
Supplement: Supplementary file 1 — Supplementary Information. [file 41598_2023_29171_MOESM1_ESM.docx]

SI Figure 4b Proxigram data

| **Distance (nm)** | **Be %** | **Fe %** | **Y %** | **Ce %** | **U %** | **Sample Count** |
| --- | --- | --- | --- | --- | --- | --- |
| **-15.25** | 0.090799 | 6.251654 | 0.090280 | 0.113110 | 0.086648 | 192733 |
| **-14.75** | 0.104750 | 6.168813 | 0.091267 | 0.114084 | 0.106824 | 192841 |
| **-14.25** | 0.107316 | 6.105045 | 0.093251 | 0.131280 | 0.094813 | 191956 |
| **-13.75** | 0.111583 | 6.386318 | 0.092812 | 0.137654 | 0.083948 | 191785 |
| **-13.25** | 0.123463 | 6.326839 | 0.098979 | 0.107835 | 0.084914 | 191960 |
| **-12.75** | 0.108320 | 6.247338 | 0.097804 | 0.134611 | 0.102536 | 190177 |
| **-12.25** | 0.101865 | 6.249837 | 0.110224 | 0.115448 | 0.089328 | 191429 |
| **-11.75** | 0.127499 | 6.306499 | 0.101895 | 0.136905 | 0.102417 | 191374 |
| **-11.25** | 0.102014 | 6.311116 | 0.106771 | 0.128971 | 0.076114 | 189190 |
| **-10.75** | 0.120927 | 6.309436 | 0.097060 | 0.136839 | 0.086452 | 188543 |
| **-10.25** | 0.106105 | 6.477728 | 0.103453 | 0.132101 | 0.095495 | 188492 |
| **-9.75** | 0.106089 | 6.346160 | 0.086364 | 0.117818 | 0.101291 | 187578 |
| **-9.25** | 0.107118 | 6.327974 | 0.105520 | 0.118843 | 0.103921 | 187643 |
| **-8.75** | 0.108396 | 6.295008 | 0.108932 | 0.118055 | 0.099273 | 186354 |
| **-8.25** | 0.128814 | 6.354829 | 0.104125 | 0.141696 | 0.095000 | 186315 |
| **-7.75** | 0.130141 | 6.337119 | 0.106479 | 0.146274 | 0.080666 | 185952 |
| **-7.25** | 0.118020 | 6.356905 | 0.117481 | 0.140115 | 0.093769 | 185562 |
| **-6.75** | 0.117418 | 6.443573 | 0.107277 | 0.147840 | 0.096603 | 187365 |
| **-6.25** | 0.132413 | 6.477988 | 0.118418 | 0.148560 | 0.097964 | 185783 |
| **-5.75** | 0.116527 | 6.438388 | 0.109578 | 0.150202 | 0.105837 | 187081 |
| **-5.25** | 0.124349 | 6.424700 | 0.119566 | 0.159422 | 0.105218 | 188180 |
| **-4.75** | 0.126774 | 6.370790 | 0.133612 | 0.161492 | 0.126774 | 190102 |
| **-4.25** | 0.153080 | 6.331773 | 0.152567 | 0.190580 | 0.120204 | 194669 |
| **-3.75** | 0.166513 | 6.373784 | 0.123250 | 0.227887 | 0.145888 | 198783 |
| **-3.25** | 0.149355 | 6.129823 | 0.151764 | 0.262094 | 0.169590 | 207559 |
| **-2.75** | 0.193965 | 5.879059 | 0.147068 | 0.328284 | 0.222195 | 219627 |
| **-2.25** | 0.209007 | 5.647356 | 0.155083 | 0.412997 | 0.300969 | 239227 |
| **-1.75** | 0.266378 | 5.474269 | 0.153264 | 0.475235 | 0.362892 | 259030 |
| **-1.25** | 0.330734 | 5.362431 | 0.178202 | 0.658864 | 0.493311 | 268796 |
| **-0.75** | 0.447541 | 5.270780 | 0.204975 | 0.866592 | 0.707123 | 252714 |
| **-0.25** | 0.672538 | 5.225213 | 0.239689 | 1.325337 | 1.112907 | 212776 |
| **0.25** | 0.963357 | 5.239838 | 0.272215 | 2.034539 | 1.937320 | 169719 |
| **0.75** | 1.182148 | 4.857833 | 0.365953 | 3.371544 | 3.625815 | 142368 |
| **1.25** | 1.144567 | 3.875830 | 0.478651 | 4.758538 | 6.126889 | 128695 |
| **1.75** | 0.842864 | 2.819026 | 0.534331 | 6.120673 | 8.519990 | 116033 |
| **2.25** | 0.513710 | 1.862197 | 0.626084 | 7.189794 | 10.257069 | 93438 |
| **2.75** | 0.413492 | 1.424669 | 0.654070 | 7.845078 | 11.582799 | 79808 |
| **3.25** | 0.284087 | 1.250791 | 0.677231 | 7.903276 | 12.703136 | 74273 |
| **3.75** | 0.217066 | 1.072964 | 0.680048 | 7.891302 | 13.470442 | 72789 |
| **4.25** | 0.126737 | 1.096070 | 0.665720 | 8.015097 | 13.825520 | 71802 |
| **4.75** | 0.127746 | 1.075314 | 0.607847 | 7.674598 | 14.153155 | 71235 |
| **5.25** | 0.076110 | 1.122982 | 0.636165 | 7.619622 | 14.341720 | 69636 |
| **5.75** | 0.061890 | 1.122658 | 0.601629 | 7.439765 | 14.785975 | 69478 |
| **6.25** | 0.044066 | 1.133960 | 0.590482 | 7.403055 | 14.734136 | 68080 |
| **6.75** | 0.044316 | 1.138932 | 0.574636 | 7.420046 | 14.891794 | 67695 |
| **7.25** | 0.039666 | 1.142692 | 0.637710 | 7.425206 | 14.883976 | 65547 |
| **7.75** | 0.027829 | 1.174997 | 0.599867 | 7.325181 | 15.063156 | 64681 |
| **8.25** | 0.035163 | 1.307440 | 0.594582 | 7.374730 | 14.870934 | 62565 |
| **8.75** | 0.039270 | 1.261556 | 0.631596 | 7.310807 | 14.955412 | 61115 |
| **9.25** | 0.022202 | 1.221137 | 0.631917 | 7.270460 | 15.078904 | 58552 |
| **9.75** | 0.036609 | 1.227272 | 0.587487 | 7.489148 | 14.985269 | 57363 |
| **10.25** | 0.023739 | 1.259998 | 0.644608 | 7.214857 | 15.039626 | 54762 |
| **10.75** | 0.020626 | 1.316308 | 0.607527 | 7.369072 | 14.910652 | 53331 |
| **11.25** | 0.017315 | 1.289032 | 0.584874 | 7.297458 | 14.952768 | 51977 |
| **11.75** | 0.027775 | 1.198294 | 0.565420 | 7.407995 | 15.212777 | 50405 |
| **12.25** | 0.029151 | 1.213926 | 0.616333 | 7.289801 | 15.123058 | 48026 |
| **12.75** | 0.023994 | 1.208446 | 0.687113 | 7.342291 | 14.917983 | 45844 |
| **13.25** | 0.013553 | 1.210752 | 0.639259 | 7.379715 | 15.231534 | 44270 |
| **13.75** | 0.045653 | 1.456101 | 0.696814 | 7.362199 | 14.935845 | 41618 |
| **14.25** | 0.019807 | 1.393909 | 0.623917 | 7.685071 | 14.959148 | 40390 |
| **14.75** | 0.021966 | 1.312466 | 0.664470 | 7.314662 | 15.038440 | 36420 |
| **15.25** | 0.014337 | 1.270287 | 0.599300 | 7.400929 | 15.234845 | 34874 |
